# Lactoferrin mitigates ethanol-induced gastric ulcer via modulation of ROS/ICAM-1/Nrf2 signaling pathway in Wistar rats

**DOI:** 10.22038/IJBMS.2022.66823.14656

**Published:** 2022-12

**Authors:** Gihan F. Asaad, Rasha E. Mostafa

**Affiliations:** 1 Department of Pharmacology, Medical Research and Clinical Studies Institute, National Research Centre, Dokki, Cairo 12622, Egypt

**Keywords:** Anti-oxidant, Anti-inflammatory, Ethanol, Gastric ulcer, Lactoferrin

## Abstract

**Objective(s):**

We aimed to investigate the gastroprotective effect of lactoferrin (LF; 100 & 300 mg/kg) in male Wistar rats *versus* gastric ulcers induced by 96% ethanol.

**Materials and Methods:**

Rats were randomly allocated into 4 groups: control, ethanol, ethanol+LF100, and ethanol+LF300. LF100 & 300 were given 15 days before ulcer induction. At the end of the experiment, the gastric mucosa was examined macroscopically and microscopically.

**Results:**

The ethanol group showed damage and degeneration of the stomach mucosa in addition to elevation of oxidative and inflammatory biomarkers. LF showed explicit healing of the gastric mucosal damage. LF reduced gastric malondialdehyde (MDA), tumor necrosis factor α (TNF-α), interleukin-1β (IL-1β), myeloperoxidase (MPO), and intracellular adhesion molecule-1 (ICAM-1). On the other hand, LF elevated the depleted reduced glutathione (GSH) and Nuclear factor-erythroid factor 2 (Nrf2).

**Conclusion:**

Our current study is the first to study the antiulcer effect of LF via its potential modulatory effects on the ROS/ICAM-1/Nrf2 signaling pathway. Moreover, we concluded that pretreatment with LF100 & 300 mitigated the ethanol-induced gastric ulcer via modulation of both oxidative stress and inflammatory responses.

## Introduction

Gastric ulcer disease deaths in Egypt reached 1,339 in 2020, accounting for 0.25 percent of all deaths, according to the latest WHO data. Egypt ranks #119 in the world with an age-adjusted death rate of 1.89 per 100,000 people (“Peptic Ulcer Disease in Egypt,” 2020). Gastric mucosal injury can be caused by stress, cigarette smoking, and alcohol consumption (2). Although the mechanisms underlying ethanol-induced stomach injury remains unknown, reactive oxygen species (ROS), pro-inflammatory mediators such as cytokines, and neutrophil infiltration are well recognized to play a role in the development of gastric ulcers (3)50 g/l NaCl or 0.3 mol/l HCl was pretreated to normal or 800 mL/l ethanol-induced acute gastritis Sprague-Dawley rats before a subsequent challenge with 500 ml/l ethanol. Both macroscopic lesion areas and histological damage scores were determined in the gastric mucosa of each group of animals. Besides, gastric mucosal activities of NO synthase isoforms and of superoxide dismutase, along with mucosal level of leukotriene (LT. Nuclear factor-erythroid factor 2 (Nrf2) is important in preventing oxidative damage in the gastrointestinal system as stomach ulcers caused by ethanol and other damaging substances (4). Heme oxygenase-1 (HO-1) is one of the anti-oxidant defense proteins induced by Nrf2 (5). It has been shown that HO-1 reacts to oxidative stress and cellular damage (6). Tumor necrosis factor-α (TNF-α) is a pleiotropic cytokine that triggers inflammatory responses via the NF-κβ signaling pathway, which is frequently accompanied by an increase in malondialdehyde (MDA) and a decrease in reduced glutathione (GSH) (7). Moreover, TNF-α, interleukin-6 (IL-6), and interleukin-1β (IL-1β) are cytokines that play a major role in the development of stomach ulcers (8). The creation of stomach lesions is linked to ethanol-induced neutrophil infiltration, a crucial step in the development of gastric ulcers, and can be measured by myeloperoxidase (MPO) activity (9). Other aspects of the inflammatory process, such as adhesion molecule expression, *viz., *intracellular adhesion molecule-1 (ICAM-1), are also critical to assess (10). ICAM-1 is an immunoglobulin (Ig)-like cell adhesion molecule that is expressed by a variety of cell types, including leukocytes and endothelial cells. It aids in the transendothelial migration of leukocytes to inflammatory areas (11). ICAM-1 is up-regulated in the vascular endothelium in response to inflammatory stimuli, which then regulates leukocyte recruitment and adherence to the endothelium during the early stages of vascular inflammation, eventually leading to the advancement of a variety of vascular disorders (12). 

Lactoferrin (LF) is an iron-binding glycoprotein with many functions. LF is present in a variety of biological fluids, including milk and neutrophilic granules (13). LF receptors are abundant in lymphocytes, platelets, macrophages, dopaminergic neurons, megakaryocytes, and endothelial cells, among other cell types. LF uptake is aided by certain of these receptors. LF is delivered through a receptor-mediated mechanism in cerebral endothelial cells, with no intra-endothelial breakdown (14). When it comes to bacterial, fungal, and viral infections, LF is the first line of protection (15). It has an anti-inflammatory impact (16), stimulates wound healing and bone formation, has anticancer properties (17), and has anti-oxidant capacity while lowering ROS production (18). As a result, it is reasonable to believe that LF could help alleviate stomach mucosal lesions induced by ethanol exposure by reducing oxidative damage, suppressing the pro-inflammatory response, and boosting gastroprotective defense. The current study aims to scout the modulatory effect of LF on the ROS/ICAM-1/Nrf2 signaling pathway in ethanol-induced gastric ulcers in rats. The histopathological investigation was used to assess the integrity of the stomach mucosa.

## Materials and Methods

***Materials and reagents***

Pravotin® (LF) was procured from Hygint Pharmaceuticals Co. (Alexandria, Egypt). Ethanol (purity *> *99%) was purchased from El Nasr Pharmaceutical Chemicals Co. (Cairo, Egypt). Spectrophotometric kits assay: MDA (Biovision Inc., Milpitas, CA, Catalog # K739-100), GSH (Biovision Inc., Milpitas, CA, Catalog # K464-100), MPO (Biovision Inc., Milpitas, CA, Catalog # K464-100). Enzyme-Linked Immuno-Sorbent Assay (ELISA) kits: Nrf2 (Abcam, Cambridge, UK, Catalog # ab207223), Interleukin 1 Beta (IL1β) (Cloud-clone Corp., TX, USA, Catalog # SEA563Ra), Rat TNF-α (Biolegend, San Diego, CA, USA, Catalog # 438204) and Rat ICAM1 (Abbexa, Houston, TX, USA, Catalog # abx155662) were used for the *in vivo *assessment of oxidative and inflammatory biomarkers in tissue. All other chemicals used in the experiments were of high analytical grades.

***Animals and experimental design***

Forty adult male Wistar rats weighing (150-200 g) were purchased from the Animal Breeding Unit at the National Research Centre -Dokki- Cairo – Egypt. Animals were housed in cages with water and food *ad libitum*. Suitable procedures were taken to minimalize the pain or discomfort of the animals. All the animals were kept in a room with a 12-hour light/dark cycle, a temperature of 22 °C, and relative humidity of 65 %. The rats were divided into four groups (n=10) at random. Group 1 served as a control group and was given normal saline for 15 days. Group 2 served as ethanol control and was given normal saline for 15 days till the day of ethanol induction. Group 3 (LF 100 mg/kg orally; 15 days). Group 4 (LF 300 mg/kg orally; 15 days). At the end of the 15 days, ethanol was given to groups (2-4) (96%, 1 ml/rat, orally) (19). Experiments were approved by the Medical Research Ethics Committee (MREC), National Research Centre, Approval Number: 3415062022, Approval Date: 12/05/2022.

***Sample preparation***

Two hours post ethanol administration, the animals were anesthetized with intraperitoneal ketamine (50 mg/kg), and their stomachs were removed and dissected at the greater curvature for ulcer scoring (ulcer number and severity) according to Sánchez-Mendoza *et al.* (20) and Abdel-Aziz *et al. *(21). Tissues were washed thoroughly and rinsed with ice. They were gently blotted between the folds of a filter paper and weighed in an analytical balance. 10% homogenate was prepared in 0.05 M phosphate buffer (pH 7) using a polytron homogenizer at 40C. The homogenate was centrifuged at 10,000 rpm for 20 min to remove the cell debris, unbroken cells, nuclei, erythrocytes, and mitochondria. The supernatant (cytoplasmic extract) was used to estimate MDA, GSH, Nrf2, TNF-α, IL-1β, MPO, and ICAM-1 according to manual instructions. Protein content in the tissue was determined according to Bradford *et al.* (22) using a Genei protein estimation kit (Bangalore, India). All ELISA kits were measured by an ELISA reader. Color absorbance was read at an OD range of 450 to 630 nm using an ELISA plate reader (Stat Fax 2200, Awareness Technologies, Florida, USA).

For histopathological studies, the stomach specimens were preserved in 10% buffered neutral formalin. The fixed tissue was rinsed in tap water, dehydrated through graded series of alcohols, cleared in xylene, and embedded in paraffin wax. 5 μm thick sections were cut and stained with hematoxylin and eosin (H&E), and then the tissues were examined and evaluated by light microscopy.

***Determination of oxidative stress biomarkers (MDA, GSH, and Nrf2)***

MDA was determined according to Ohkhawa *et al*. (23). The method is based on the interaction of thiobarbituric acid (TBA) with MDA in an acidic medium at 95°C for 30 minutes to produce the TBA reactive product. The MDA produces colorful complexes. Absorbance was determined spectrophotometrically at 532 nm.

GSH was determined according to Ellman (24). The method is based on reducing 5,5-dithiobis-2-nitrobenzoic acid to produce a yellow compound. The reduced chromogen is directly proportional to GSH concentration, and its absorbance can be measured calorimetrically at 405 nm.

Nrf2 transcription factor levels were measured using a commercially available ELISA kit (BT-LAB). Microplates coated with human Nrf2 antibodies are employed in this procedure. These plates are then filled with stomach homogenate and incubated. After washing the plates, the secondary antibody Streptavidin-horseradish peroxidase is added and incubated once more. Color is created after the following washing with the addition of substrate. The reaction is halted with a stop solution, and the absorbance is measured on a microplate reader at 450 nm. (Thermo-Go). 

***Determination of inflammatory biomarkers (TNF-α, IL-1β, ICAM-1, and MPO)***

TNF-α, IL-1β, and ICAM-1 were measured using commercial ELISA kits. Stomach homogenate (100 μl) was added to a 96-well plate coated with an antibody specific to the inflammatory marker at room temperature for 2 hr and incubated with diluted biotin-conjugated antibody (100 μl) at 37 °C for 1 hr. Avidin-Horseradish peroxidase (HRP) conjugate (100 μl) was added to the 96-well plate at 37 °C for 30 min with several washes in between and reacted with TMB chromogenic reagent (100 μl) in the dark for 15–30 min. The reaction was terminated by adding sulphuric acid solution (100 μl), and the absorbance was determined at 450 nm.

Gastric MPO activity was measured spectrophotometrically using a commercial kit. Stomach homogenate (50 μl) was mixed with assay buffer (50 μl) in the dark at 25 °C. After 30-, 60-, and 120-min incubation, stop solution (2 μl) was added for 10 min, followed by adding the chromophore 5-thionitrobenzoic acid (TNB) (50 μl). The absorbance was measured at 412 nm. One unit of MPO activity is defined as the amount of MPO for producing taurine chloramine to react with 1.0 μmole of TNB per min at 25 °C.

***Statistical analysis***

Data were presented as mean ± standard error. Statistical analysis was done by one-way analysis of variance (ANOVA) followed by Tukey’s *post hoc*. The non-parametric examinations were done using the Kruskal-Wallis non-parametric ANOVA test, proceeded by the Mann-Whitney U test. *P*˂0.05 was considered to signify the statistical difference. (Graph Pad Software, Inc., San Diego, CA, USA) was used to carry out all statistical tests. 

## Results

***Ulcer number and severity***

**Figure 1:**
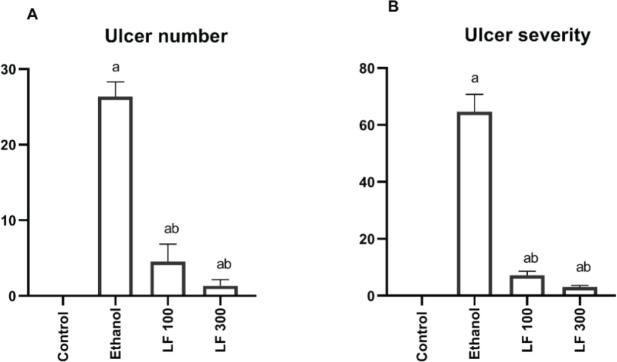
Effect of lactoferrin (LF, 100 & 300 mg/kg) vs. ethanol-induced stomach ulcers on ulcer number (A) and ulcer severity (B). The data is presented as mean ± standard error (n = 10)

As rats were given 96 % ethanol orally (1 ml/rat), both the number of ulcers and the severity of the ulcers increased significantly (*P<*0.05) when compared to the control group (26.33 ± 0.8, 64.67 ± 6.08). In comparison to the ethanol group, rats given LF at two dose levels (100 & 300 mg/kg; orally) for 15 days before induction of gastric ulcer showed a significant (*P<*0.05) ameliorating effect against ethanol-induced gastric ulcer by abolishing ulcer number (4.5 ± 0.6, 1.3 ± 0.3, respectively) and ulcer severity (7.16 ± 1.4, 3.0 ± 0.63, respectively). LF 100 and 300 administration resulted in an inhibition of gastric ulcer severity of 88.92% and 95.36%, respectively, demonstrating an unambiguous preventive effect against produced gastric ulcer. The results are depicted in Figure 1. 

***Assessment of oxidative biomarkers (MDA, GSH, and Nrf2)***

**Figure 2:**
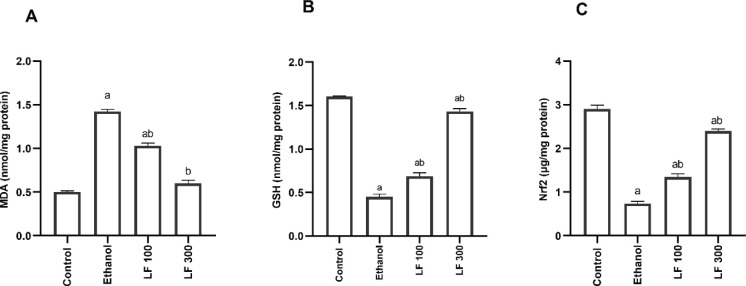
Effect of lactoferrin (LF, 100 & 300 mg/kg) vs. ethanol-induced stomach ulcers on MDA (A) and GSH (B) and Nrf2 (C). The data is presented as mean ± standard error (n = 10). GSH; Reduced glutathione, MDA; Malodialdehyde, Nrf2; Nuclear factor-erythroid factor 2

In comparison to the control group (0.5 ± 0.01 nmol/mg protein), the ethanol group had a substantial (*P<*0.05) rise in MDA concentration (1.42 ± 0.02 nmol/mg protein) of 184 %. Ethanol, on the other hand, significantly (*P<*0.05) reduced GSH and Nrf2 levels (0.45 ± 0.03 nmol/mg protein & 0.74 ± 0.05 μmol/mg protein), with a reduction of 71.87 % and 74.5 %, respectively, when compared to the control group (1.6 ± 0.007 nmol/mg protein & 2.9 ± 0.09 μmol/mg protein). When compared to the ethanol-control group, oral LF (100 mg/kg) treatment for 15 days before ethanol administration significantly (*P<*0.05) lowered the induced MDA (1.03 ± 0.03 nmol/mg protein), suggesting a 27.46 % reduction. In contrast to the ethanol-control group, LF100 generated a significant (*P<*0.05) increase in depleted GSH and Nrf2 (0.69 ± 0.042 nmol/mg protein & 1.35 ± 0.067 μmol/mg protein) with an increase of 53.33 % and 82.43% respectively. After 15 days of oral LF (300 mg/kg) treatment prior to ethanol delivery, MDA concentration (0.6 ± 0.03 nmol/mg protein) was considerably (*P<*0.05) restored. In contrast to the ethanol-control group, LF300 induced a substantial (*P<*0.05) rise in depleted GSH and Nrf2 (1.44 ± 0.029 nmol/mg protein & 2.4 ± 0.044 μmol/mg protein) with a percent increase (220 % and 224.32 %) respectively. LF, as a result, has been found to exert stomach-protective properties in a dose-dependent way. The results are represented in Figure 2.

***Assessment of inflammatory biomarkers (TNF-α, IL-1β, MPO, and ICAM-1)***

Ethanol 96% (1ml/rat) caused a significant increment in stomach tissue TNF-α, IL-1β, MPO, and ICAM-1 levels to 312.39%, 608.83%, 173.68%, and 326.08%, respectively, compared to the control group. Oral LF treatment (100 mg/kg body weight) for 15 days before ethanol induction lowered the elevated stomach tissue pro-inflammatory biomarkers levels to 35.09%, 43.33%, 15.36%, and 31.43%, respectively, compared to the ethanol-control group.

**Figure 3:**
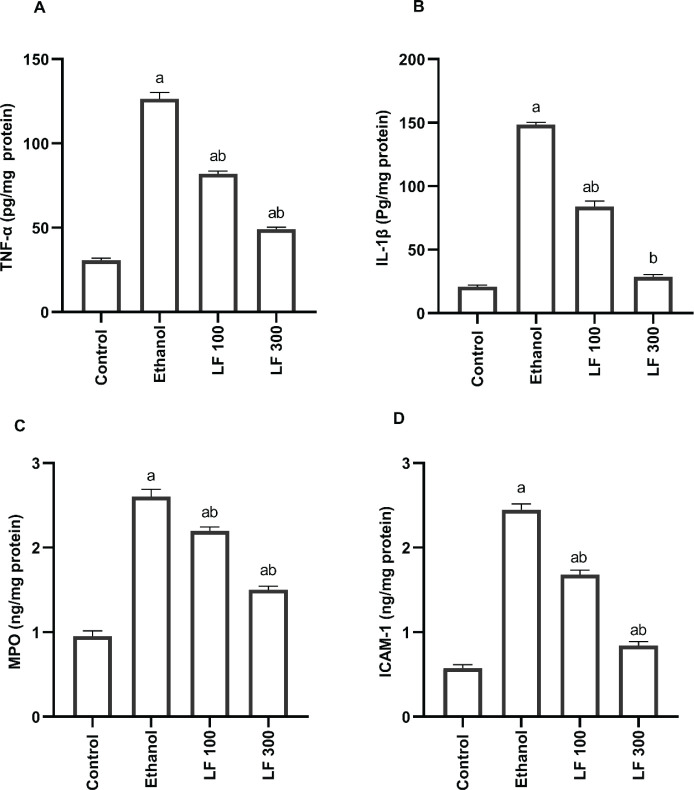
Effect of lactoferrin (LF, 100 & 300 mg/kg) vs. ethanol-induced stomach ulcers on TNF-α (A) and IL-1β (B), MPO (C) and ICAM-1 (D). The data is presented as mean ± standard error (n=10). TNF-α; Tumor necrosis factor α, IL-1β; Interleukin 1β, MPO; Myeloperoxidase, ICAM-1; intercellular adhesion molecule 1

Oral LF treatment (300 mg/kg body weight) for 15 days before ethanol induction lowered the elevated stomach tissue pro-inflammatory biomarkers levels to 61.12%, 80.71%, 42.31%, and 65.71%, respectively, compared to the ethanol-control group. The results are represented in Figure 3.

***Macroscopical findings***

**Figure 4:**
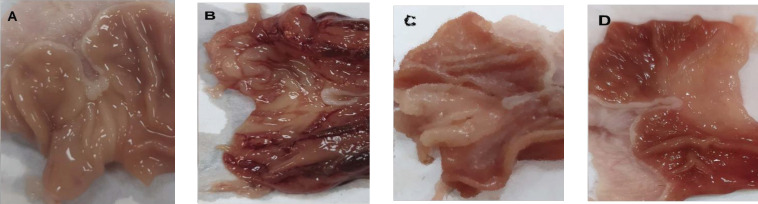
Macroscopic examination of gastric tissues of rat where A, a photomicrograph of the gastric tissue of a control group; B, a photomicrograph of the gastric tissue of the ethanol group; C, a photomicrograph of the gastric tissue of Ethanol + LF 100 and D, a photomicrograph of the gastric tissue of Ethanol + LF 300

No hemorrhagic lesions or mucosal erosions were observed during the macroscopic examination of the stomach tissue of the control group (Figure 4A). The ethanol group showed significantly intensified gastric lesions during the macroscopic examination compared to the control group (Figure 4B). The groups administered LF100, and LF 300 showed minute mucosal injury with less superficial disruption in the gastric epithelial lining (Figure 4C and Figure 4D).

***Histopathological findings***

**Figure 5:**
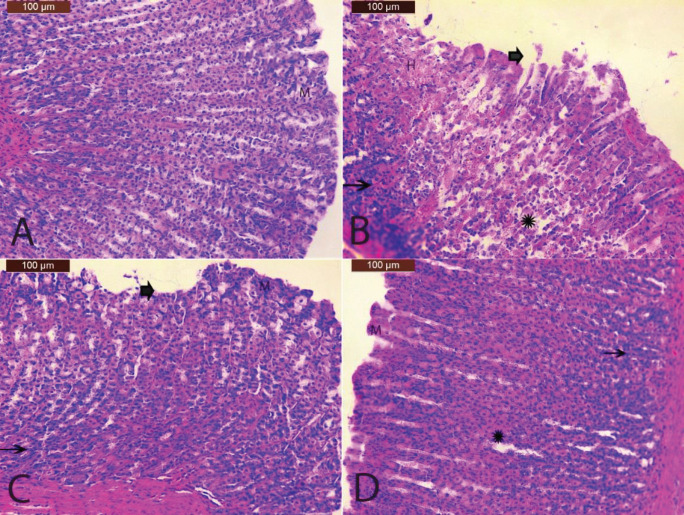
Microscopic histopathologic examination of gastric tissues of tatwhere the letter (M) indicates intact stomach structure. The thick arrow indicates degeneration, erosions in the superficial layer of the mucosa, and disruption to the deep mucosa layer. Star indicates edema. The thin arrow indicates visible inflammatory cells. Letter (H) indicates hemorrhagic necrosis of the mucosa. LF; Lactoferrin (H&E, x200)

Microscopic examination of the control group revealed a normal histological appearance of the intact stomach structure (mucosa, muscularis mucosae, submucosa) (Figure 5A). The ethanol group showed damage and remarkable lesions such as degeneration, erosions of the surface epithelial layer of the stomach mucosa, and disruption to the deep mucosa layer. Also, edema, hemorrhagic and inflammatory cells visible in stomach mucosa were observed (Figure 5B). The group treated with ethanol and LF 100 had mild damage to the surface epithelium and no edema and mild inflammatory cells (Figure 5C). In addition, the group treated with ethanol and LF 300 have relatively better protection, as observed by decreasing ulcer area and reduced or complete absence of edema and inflammatory cells (Figure 5D). LF has been shown to exert protective effects on the stomach in a dose-dependent manner. 

a *P*<0.05: significantly different compared to the control group

b *P*<0.05: significantly different compared to the ethanol group

a *P*<0.05: significantly different compared to the control group

b *P*<0.05: significantly different compared to the ethanol group

a *P*<0.05: significantly different compared to the control group

b *P*<0.05: significantly different compared to the ethanol group

## Discussion

In the current study, our results showed that oral administration of 96% ethanol (1 ml/rat) caused the development of intensified gastric lesions confirmed by histopathological degeneration, erosions of the surface epithelial layer of the stomach mucosa, and disruption to the deep mucosa layer. In addition, edema, hemorrhagic and inflammatory cells are visible in the stomach mucosa. In a recent study, similar results have been reported by El-Dakroury *et al.* (19). Reactive oxidants are created in many compartments inside the cell from a variety of sources, either naturally or as a result of toxic or pathologic assaults (25). Previous research demonstrated that ethanol exposure causes the development of ROS, which further causes stomach injury. Therefore, enhancing the anti-oxidant signaling can protect the gastric mucosa against ulceration (26). In agreement with a previous study, our results showed that ethanol administration elevated MDA levels to 184%, and on the other hand, it depleted the levels of both GSH and Nrf2 to 71.87 % and 74.5 %, respectively. Pretreatment with LF at both dose levels (100 and 300 mg/kg) for 15 days showed a significant recovery of the gastric mucosal lesions as well as alleviation of the histopathological findings. To maintain redox homeostasis in the cell, ROS are counterbalanced by complex anti-oxidant mechanisms. Low-molecular-weight anti-oxidants, such as GSH, are important anti-oxidants (27). Also, it has been reported that pharmacological boosting of the Nrf2 activity with chemoprotective agents protected animals from oxidative damage (28). Sudden induction of Nrf2 happens when endogenous or external cellular events requiring cellular defense systems, such as oxidative damage and/or inflammation, occur (29). LF at both dose levels caused a substantial reduction of MDA and a prominent elevation of the endogenous GSH and Nrf2. These results could be attributed to the anti-oxidant effect of LF, as it has been noted that LF is a significant specialized iron scavenger and that its anti-oxidant activity is most likely due to its capacity to bind ferrous and ferric ions. Being an iron scavenger, LF may prevent the Fenton reaction that describes the formation of hydroxide (OH^−^) and hydroxyl radical by a reaction between Iron (II) (Fe^2+^) and hydrogen peroxide (H_2_O_2_) (30). 

Cytokines are small proteins and polypeptides generated by a variety of cells that govern cell development, differentiation, and immunological function, as well as inflammation and wound healing. Interleukins (ILs), interferons, tumor necrosis factor (TNF), MPO, and ICAM-1 are examples of cytokines (31). Oxidative damage occurs in target cells when cytokines are overproduced in response to oxidative stress. Several pro-inflammatory cytokines are overproduced when NF-κβ is activated by oxidative stress. Furthermore, pro-inflammatory oxidative stress leads to increased NF- κβ activation and cytokine overproduction (32). MPO is a crucial inflammatory enzyme and therapeutic target that triggers both oxidative stress and inflammation. It is found in infiltrated neutrophils. Its activation can catalyze the release of ROS, altering inflammatory signaling pathways (33). At sites of inflammation, ICAM-1 expression is greatly elevated, providing a key mechanism for regulating cell-cell interactions and, presumably, inflammatory responses. In a variety of experimental animal models, the role of leukocyte adhesion in the production and maintenance of inflammation has been established (34). In the current study, ethanol elevated the pro-inflammatory cytokines TNF-α, IL-1β, MPO, and ICAM-1 to 312.39%, 608.83%, 173.68%, and 326.08%, respectively. Similar results have been reported by Liu *et al*. (35) and Lian *et al*. (36). TNF-α, IL-1β, MPO, and ICAM-1 levels in the gastric homogenate were significantly lowered in groups pretreated with LF 100 and 300. As per our findings, LF possessed anti-inflammatory effects by lowering gastric pro-inflammatory indicators. Our findings also revealed that the impact of LF increased in a dose-dependent manner. We assumed that anti-inflammatory actions of LF were related to the elevation of Nrf2. In a previous study on human retinal pigment epithelial cells, TNF-α induced ICAM-1 expression was shown to be inhibited by overexpression of Nrf2 (37). Hence, this suggests that Nrf2 plays a crucial role in the inflammatory process by regulating the migration and infiltration of inflammatory cells into inflamed tissue. We strongly recommend further studies to add LF to conventional anti-gastric ulcer medicines based on our findings, which show that LF exerts anti-oxidant and anti-inflammatory actions by targeting the ROS-ICAM-Nrf2 signaling pathway. 

## Conclusion

LF, at both dose levels, was able to protect the gastric mucosa against ethanol-induced gastric ulcers that mimicked ulcers in humans through multiple mechanisms of action, including the anti-oxidant effects (reduction of MDA and elevation of GSH and Nrf2 levels) and anti-inflammatory effects (reduction of pro-inflammatory cytokines; TNF-α, IL-1, MPO, and ICAM-1). As a result, LF has the potential to be used as preventative and supplementary medicine to help prevent the formation of stomach ulcers and recurrence episodes. Further studies are warranted. Finally, we attribute the anti-gastric ulcer of LF to its modulatory effect on the ROS-ICAM-Nrf2 signaling pathway.

## Authors’ Contributions

The manuscript was conceptualized, investigated, supervised, and written with the help of Gihan F. Asaad and Rasha E. Mostafa. The published version of the manuscript has been read and approved by all authors.

## Funding

The authors stated that they did not receive any funding for their study.

## Conflicts of Interest

The authors declared that there is not any conflict of interest.
